# MicroRNA as a Novel Biomarker in the Diagnosis of Head and Neck Cancer

**DOI:** 10.3390/biom11060844

**Published:** 2021-06-05

**Authors:** Jacek Kabzinski, Monika Maczynska, Ireneusz Majsterek

**Affiliations:** Department of Clinical Chemistry and Biochemistry, Medical University of Lodz, al. Kościuszki 4, 90-419 Łódź, Poland; jacek.kabzinski@umed.lodz.pl (J.K.); monika.maczynska@umed.lodz.pl (M.M.)

**Keywords:** cancer, HNSCC, microRNA, miRNA, biomarker

## Abstract

Head and neck squamous cell carcinoma is the sixth most common cancer worldwide, with 890,000 new cases and 450,000 deaths in 2018, and although the survival statistics for some patient groups are improving, there is still an urgent need to find a fast and reliable biomarker that allows early diagnosis. This niche can be filled by microRNA, small single-stranded non-coding RNA molecules, which are expressed in response to specific events in the body. This article presents the potential use of microRNAs in the diagnosis of HNSCC, compares the advances in this field to other diseases, especially other cancers, and discusses the detailed use of miRNA as a biomarker in profiling and predicting the treatment outcome with radiotherapy and immunotherapy. Potential problems and difficulties related to the development of this promising technology, and areas on which future research should be focused in order to overcome these difficulties, were also indicated.

## 1. Head and Neck Cancer

Head and neck cancers (HNC) are a group of heterogeneous diseases, including tumors formed most often from the epithelial cells (in 85% of cases) of the laryngeal and oropharynx, lips, mouth, nasopharynx or larynx, all of which are different in their self-epidemiology, etiology and method of treatment. Head and neck cancers account for about 6% of all cancers and cause about 1–2% of deaths due to neoplastic diseases [1,2,3]. Further, 90% of all HNCs are squamous cell carcinomas (HNSCCs), arising from the mucosal lining in these regions. HNSCC is the sixth most common cancer worldwide, with 890,000 new cases and 450,000 deaths in 2018 [4,5]. The incidence of HNSCC continues to rise and is anticipated to increase by 30% by 2030 according to the Global Cancer Observatory.

Exposure to carcinogens, diet, oral hygiene, infectious agents and other diseases, individually and in combination, may influence the risk of developing HNSCC [6]. Smoking tobacco products is an independent risk factor for head and neck cancers. The risk increases with the duration of the addiction and its intensity [7]. Smoking cessation reduces, but does not completely eliminate, the risk of HNSCC. Passive exposure to cigarette smoke also increases the chance of developing cancer. Alcohol is another of the independent factors that lead to cancer development. It has a synergistic effect with the effects of tobacco smoke. Alcohol in the human body is metabolized into acetaldehyde. This compound creates adducts with the DNA, thus damaging the cell’s DNA [8]. Human papillomavirus, especially the highly oncogenic HPV16 type, is responsible for the development of squamous cell tumors of the oropharynx. HPV16 and 18 genomic DNA is found in 25% of HNSCC cases. Studies published in 2006–2009 show that HPV16 is responsible for approximately 55% (out of 654 taken into account in the study) of squamous nasopharyngeal neoplasms [9]. The hereditary diseases that increase the risk of HNSCC include the following: Fanconi’s anemia (FA), ataxia, telangiectasia, Bloom’s syndrome and Li-Fraumeni syndrome [10]. Fanconi anemia is an autosomal recessive or X-linked disease, predisposing to the development of solid tumors and leukemias. The mutation concerns one of the 15 FA genes involved in DNA repair. HNSCC is one of the most commonly diagnosed solid tumors in FA patients, and the risk of developing cancer is 800 times higher than that of the general population. Tumors develop in patients with this disease much earlier than in the general population. HNSCC in these patients develops mainly in the oral cavity [11]. Other congenital diseases that predispose to HNSCC include congenital immunodeficiencies. People with impaired or reduced immunity (immunosuppression) from malnutrition, the elderly, and those who have had a transplant or have AIDS have a higher risk of developing cancer. The most common cancers in the latter are Kaposi’s sarcoma and non-Hodgkin’s lymphoma, but there is also a higher risk of developing oropharyngeal squamous cell carcinoma [6]. As with other cancers, the risk of developing HNSCC increases with age. Most cancers affect people over 50 years of age. The mean age of HNSCC development is 78 years for non-smokers, 60 years for tobacco smokers, 58 years for HPV-positive men and 61 years for HPV-positive women [12].

## 2. MicroRNA

MicroRNAs (MiRNAs) are short (about 22 nucleotides), non-coding RNAs that are involved in the post-translational regulation of gene expression. So far, it has been found that they regulate up to 60% of mRNA through participation in the cell cycle, apoptosis, proliferation and even the cell’s response to stress [13]. Pathological changes of the above processes occur at every stage of neoplasm. Given this information, microRNA analysis is performed on every plane. About 2,000 microRNAs are encoded in the human genome, but not all of them have been described so far [14]. The first studies concerned the analysis of miRNAs in chronic lymphocytic leukemia B (PBL-B); the influence of microRNAs on the role of these molecules as key regulators of both suppressor genes and oncogenes was noticed [15]. MicroRNA is able to play such roles in tumorigenesis. The expression of a specific microRNA is not only typical for particular tissues of the organism, but also for specific tumors of various origins. The determination of the miRNA profile for individual types of neoplastic tumors determines their characteristics and indirectly may indicate the clinical and pathological features of the changes, such as the degree of tumor differentiation, the ability to angiogenesis, proliferation and migration of neoplastic cells [16]. Recent research is focused on the role of microRNA as a factor supporting the determination of the surgical margin in surgical treatment, and markers helpful in diagnostics are sought [17]. An important element is the stability of the microRNA in the analyzed material. This makes the material easy to obtain. The presence of microRNA in the blood serum has been found in hematological neoplasms and solid tumors of various origins. This may mean a straightforward path to early diagnosis of the degree of development of the neoplasma process. The presence of microRNA in body fluids as signaling molecules activating cell receptors was also indicated, which confirms also other tasks of miRNA in intracellular mechanisms, not only the regulation of genes at the post-transcriptional level. MiRNAs can function in a cancer cell as oncogenes or suppressor genes [18]. An example is miR-221 acting as a suppressor gene in erythroblastic leukemia, in contrast to solid tumors where it is an oncogene [19].

## 3. MicroRNA as an Oncogene and Protooncogene

In the transformed cell, the mechanism of control of the correct gene expression is impaired. While miRNA in a normal cell affects a given oncogene by inactivating it, in the case of the deletion of the microRNA gene, the oncogene product undergoes increased production [20,21]. On the contrary, excessive amplification of the microRNA gene that regulates the tumor suppressor causes its blockade and also opens the way to carcinogenesis. MicroRNAs can function either as oncogenes or as genes for tumor suppression [22]. The first microRNAs reported as a suppressor were miR-15o and miR-16a, located in arm 13q 14, a region that is found in more than 50% of chronic leukemia patients. MicroRNA is deleted in the pathogenesis of squamous head and marrow neck carcinomas (B-CLL) [23]. These are the only known genes in a given fragment, and what is more, they are involved in the regulation of the anti-apoptotic BCL-2 gene. All of the B-CLL patients had no or a very low expression of iniR-15a and miR-16a. Typically, neoplasms show reduced expression of miRNA genes, which may predispose to uncontrolled disease development [24]. Another example is the specifically lost expression of miR-126, which enhances tumor growth and proliferation in lung and bone cancers, and niiR-335 enhances metastasis and is its marker in breast cancer. In turn, ef-7 is a regulator of the well-known RAS oncogene (mutated by 15–30° in all tumors), which is responsible for cell growth and differentiation. In lung cancers, when RAS is overexpressed, low let-7 expression is also observed, and thus uncontrolled growth and development of lung tumors are also observed [25].

## 4. MicroRNA as a Biomarker

The symbolic date of the discovery of miRNAs is 1993, when Lee published his article [26]. Only 10 years had to pass from that moment for the first reports on the use of this discovery in diagnostics to appear. The first disease in which a biomarker potential was noticed was chronic lymphocytic leukemia, and the discovery was based on the indication that the expression of miRNA in patients is significantly different from that in healthy individuals [23]. From that moment on, intensive research into the use of miRNA as a biomarker in a wide range of diseases began. Over time, miRNA has emerged as having the potential to be an excellent biomarker as it meets three basic criteria that can be required for a quick and accurate diagnostic process. First, it is synthesized quickly in response to a pathological situation. Second, it is highly specific. Third, it remains in the system for a long time and is easily detectable due to its presence in the plasma [27]. Over time, research has focused on three main groups of diseases in which miRNA could be used as a biomarker. The first group consists of cardiovascular diseases, where the need for a new reliable marker is particularly high, as there is no gold standard for diagnosing these diseases so far. The existing markers show tissue specificity and require narrow time windows in the determinations, which often makes the obtained results unrecognizable or even false [28,29,30]. MiRNA seems to solve all these problems, hence the large-scale and advanced research towards its use as a biomarker in cardiovascular diseases [31,32,33,34]. The second group of diseases that is studied particularly intensively consists of infectious diseases. In this case, special emphasis is placed on the speed of the diagnostic process and its high specificity, which allows to think of miRNA as a potential biomarker for point of care diagnostics [35]. In addition, the presence of miRNAs in, e.g., sputum, allows for large-scale screening [36]. So far, attempts have been made to identify specific miRNAs for, inter alia, HIV [37,38,39], tuberculosis [40,41,42], malaria [43,44,45] or Ebola [46,47,48]. The latest developments in the use of miRNAs in the diagnosis of infectious diseases include, of course, research on COVID-19 [49,50]. Neoplastic diseases are the third particularly studied group of diseases. In this case, special emphasis is placed on the search for biomarkers allowing for the early differentiation of various types of cancer, which is often a problem with traditional diagnostic methods [51]. The second very promising line of research in cancer is the use of miRNAs for profiling and predicting treatment responses [52,53,54].

### Limitations

Despite all the advantages of using miRNA in diagnostics, this method has still not found its way to wide application. A number of challenges that face the implementation of each new method for general use in this case include, first of all, the need to establish unquestionable relationships between the studied miRNAs and the occurrence of a given disease, establishing guidelines for sampling and analysis, and the standardization of procedures [55,56]. The first steps taken to introduce miRNA for widespread use in diagnostics resulted in the launch of the miRNA panel in 2012 by Rosetta Genomic, allowing the identification of cancers of unknown or uncertain primary origin, followed by another panel that relied on qRT-PCR with improved sensitivity and specificity in 2016 [57]. Unfortunately, two years later the company went bankrupt and the products were withdrawn from the market [58]. Other implementation attempts are the pancreatic cancer testing panel developed by Interpace Diagnostics in 2015 by Interpace Diagnostics [59], or Mintrex with a panel using miR423-5p as a useful marker of heart failure [58]. All panels brought to the market face the difficulties of it being only a decade since the first studies showing differences in the miRNA profile between patients and healthy subjects, meaning there are no definitive answers and established procedures for controlling the pre-processing of miRNA detection and normalization experiments, data processing and optimization. The normalization strategy seems to be particularly important here [60,61], and the fact that miRNA levels can be affected by factors such as age, gender, sex, physical activity or smoking should be taken into account [62,63,64,65]. Moreover, the factor significantly influencing the miRNA profile turned out to be diet, in which various components, such as curcumin, proanthocyanidins, epigallocatechin and resveratrol, modulate the miRNA expression level, which must be taken into account when using miRNA as a diagnostic tool [66,67,68,69]. An additional potential challenge is to obtain profiles of unquestionable specificity, which, due to their uniqueness for a given disease, will leave no doubt as to the result of the diagnostic test. The coexistence of elevated levels of specific miRNA types in various diseases can lead to misdiagnosis, for example, if the same type is overexpressed in hepatocellular carcinoma as well as in Hepatitis B infection [70], in addition, this level is also altered by the use of drugs during therapy in chronic hepatitis C [71]. Therefore, only the establishment of standardized procedures will allow the introduction of a wider range of diagnostic tests to the market.

## 5. MicroRNA in Cancer Diagnostics

The detailed analysis of the miRNA profile of a wide variety of cancers has shown that specific miRNA types are deregulated with the onset of neoplastic transformation. At a later stage of the study, links with tumor classification, progression, prognosis and response to treatment were demonstrated [72,73,74]. This can be considered a unique profile for a given cancer disease, and can be referred to as an miRNA fingerprint. Due to the fact that neoplasms may originate from various types of cells, as well as may arise as a result of various pathological mechanisms, it should be expected that there will be a wide spectrum of cancers differing in terms of both clinical and genetic characteristics. In this case, an accurate diagnosis is extremely important for appropriate and effective treatment. Therefore, using miRNA fingerprint, a more accurate diagnosis can be expected than in the case of traditional methods. For many years, miRNA has been successfully linked with the diagnosis of specific types of cancer such as lung cancer [75,76], breast cancer [77,78], colorectal cancer [79,80], ovarian cancer [81,82], or cervical cancer [83,84]. Currently, however, research is going much further, allowing for far-reaching identification with a high degree of specialization. MicroRNAs may be used in identifying the tissue in which cancers of unknown primary origin arose; Rosenfeld et al.’s classification accuracy reached 100% for most tissue classes, including 131 metastatic samples [85]. Lu et al. was able to successfully classify poorly differentiated tumors using miRNA expression profiles, whereas messenger RNA profiles were highly inaccurate when applied to the same samples [86]. MicroRNA can also be used to classify a specific tumor phenotype, such as in breast cancer, where it has been possible to connect the miRNA profile with the estrogen and progesterone receptor status, proliferation and tumor stage [87,88], and even define the molecular subtype (luminal A, luminal B, basal-like, HER2 and normal-like) [89]. Further research allowed the use of miRNAs to distinguish ductal carcinoma in situ and in invasive ductal carcinoma, and thus predict the level of proliferation and aggressiveness of breast cancer [90]. For lung cancer, the miRNA expression patterns differ between non-small-cell lung carcinoma and small-cell lung carcinoma, as well as their subtypes [91,92,93]. Further, it is possible to distinguish between adenocarcinoma and squamous cell carcinoma, and in some cases even indicate the cancer stage [94,95]. In the case of leukemia, miRNA studies have shown not only the possibility of effective early disease identification [96,97], but also the distinction between chronic and acute forms [98], explaining the aggressiveness of the disease using the B-cell receptor signaling mechanism [99], and even the prediction of specific cytogenetic abnormalities that have prognostic implications allowing to identify patients with the 17p and 11q deletions, who experience the aggressive form of the disease, and patients with the 13q deletion or normal cytogenetic profiles, who experience the indolent form [100].

Another aspect of using miRNA as a biomarker in cancer is the prognosis of the treatment outcome, a field that has also been very successful. In the case of diffuse large B-cell lymphoma, high miR-21 expression was associated with relapse-free survival [101]. The levels of four miRNAs were significantly associated with overall survival in non-small-cell lung cancer patients [102], while others are associated with poor survival [103]. For pancreatic cancer, it is possible to forecast not only general prognosis, but also a detailed outcome for a specific type of treatment using gemcitabine [104]. Patients with hepatocellular carcinoma tumors had low miR-26 expression and were corelated with shorter overall survival, but at the same time had a better response to adjuvant therapy with interferon alfa [105]. Finally, miRNAs can also potentially be used to evaluate the efficacy of chemotherapeutic and surgical tumor removal treatments as it allows the assessment of tumor-specific levels of miRNA expression. Wong et al. specified that not only miR-184 levels were significantly higher in tongue SCC patients in comparison with normal individuals, but moreover the levels were significantly reduced after the surgical removal of the primary tumors [106]. In the case of colorectal cancer, miR-17-3p and miR-92, identified as markers, were significantly reduced after surgery [107]. In a study investigating miR-500 as a potential human hepatocellular carcinoma marker, its levels in sera returned to normal after the surgical treatment [108]. At the same time, however, when considering the potential benefits of using miRNAs for such advanced profiling of neoplastic diseases, one cannot forget about the limitations mentioned in the previous paragraph. One study indicates that many of the miRNAs tested may turn out to be highly nonspecific and easily lead to a misdiagnosis between breast, colorectal, lung, thyroid and melanoma tumors [109].

The final issue that should be taken into account when considering miRNA as a biomarker in cancer is the transcriptome differences between animals and humans. The standard research route is to test the hypotheses in an animal model and extrapolate the results in the human system; however, such results are not always directly transferable between species [110,111]. As demonstrated in the miRNA studies with B-cell chronic lymphocytic leukemia and B-cell non-Hodgkin lymphomas, the results should be specific to a human model to ensure diagnostic and therapeutic use [112,113].

## 6. MicroRNA in HNSCC Diagnostics

### 6.1. Onco-MiRNAs and Tumor Suppressor MiRNAs

HNSCC is an interesting type of cancer to exploit the advantages of using miRNAs as novel diagnostic tools. Due to the high diversity within HNSCC and the need for early and reliable diagnosis, miRNA has been studied and evaluated for a long time as a potential aid in cancer identification, treatment prognosis and assessment of its effectiveness. The first aspect worth noting is the definition of the role of miRNAs as oncogenes and suppressor genes in HNSCC. The initial research in this field took place more than a decade ago and confirmed that miR-21 is a putative oncogenic microRNA in head and neck cancer [114]. Oncomir is an miRNA associated with cancer and can be linked with carcinogenesis, malignant transformation and metastasis. Some oncomir genes are oncogenes and their overexpression leads to cancerous growth, while others are considered tumor suppressors, so that the underexpression of the gene leads to cancerous growth [115]. MicroRNAs can act as oncomirs responsible for the following biological processes: proliferation, migration, and angiogenesis [116]. Oncomirs are responsible for the regulation of the carcinogenesis process by activating signaling pathways. Oncomir thus increases the initiation and progression of the tumor [117]. Individual types of microRNA may influence the oncogenic mechanisms in head and neck cancers. For example, microRNA-125a is responsible for the increased proliferation and migration of cancer cells by inhibiting the expression of the p53 protein. MicroRNA-134 influences oncogenicity and metastasis by inhibiting the expression of the WWOX gene. MicroRNA-134 inhibits E-cadherin expression and promotes cell progression by targeting programmed cell death 7 (PDCD7) [118,119,120,121]. Tumor suppressor miRNA expression was often reduced in tumor samples. The let-7 microRNA group controls normal cell development and differentiation, and the reduction in let-7 contributes to carcinogenesis. The let-7 group is a group of tumor suppressors in various types of cancer, including head and neck cancers [122]. The expression of the let-7 group genes is reduced in patients with head and neck tumors, among them it was shown that let-7i most significantly suppresses the expression of the chromatin modifier, AT-rich, interacting 3B domain (ARID3B) [123]. The downregulation of microRNA-101 is upregulated by the oncogene Zeste homolog 2 (EZH2), which downregulates another rap1GAP tumor suppressor gene, promoting head and neck tumors. EZH2 is a histone methyltransferase belonging to the PRC2 group, which facilitates the trimethylation of H3K27 on the rap1GAP promoter in order to suppress its activation [124]. Reduced levels of microRNA-29 occur in head and neck tumors. MicroRNA-29b inhibits the inhibition of three beta DNA methyltransferases, which causes invasiveness by restoring E-cadherin expression through the demethylation of the promoter region [125]. In Table 1, selected miRNAs, affected genes and molecular mechanisms of action in HNSCC are presented. The miRNAs listed in the table should be treated as oncogenes or suppressors due to their interaction with one specific gene. However, a more advanced issue is the effect of miRNAs not only on a single gene, but on the entire signaling pathway. An example here would be deregulation of the PI3K/AKT signaling pathway transduction via p-AKT by miR-365a-3p in laryngeal squamous cell carcinoma (LSCC) [126]. The results indicating that miR-365a-3p may act as an oncomir, and may promote growth and metastasis in LSCC via the PI3K/AKT signaling pathway, shed new light on the intricacy of the processes in which miRNAs are involved and the complexity of the intracellular interactions, while at the same time indicating the mechanism that may lead to pathogenesis and thus indicating a potential therapeutic target for the treatment of LSCC.

### 6.2. MicroRNA as Prognostic Marker

Even neoplasms of the same type usually show large genetic diversity, which is often overlooked in treatment planning, and may result in the selection of inappropriate and thus ineffective therapy. The possibility of effective and, above all, very precise identification of the type of cancer allows for the best selection of individual therapy for the patient, and significantly increases the chances of a cure [134,135,136]. A variant allele in the KRAS 3’ untranslated region, which arises in the let-7 miRNA complementary site, was associated with disease occurrence and patient survival in HNSCC, showing significantly reduced survival time and suggesting that this variant may alter the phenotype or therapeutic response of this disease [137]. The tumor suppressor protein p53, one of the most common altered proteins in cancer resulting from the TP53 gene mutation, was evaluated for survival rate in patients with squamous cell carcinoma of the head and neck, and revealed decreased overall survival with even stronger association with disruptive mutations [138]. Further p53 studies confirmed these reports and refined the data by adding that this association was stronger in the clinical subgroup of patients subjected to adjuvant therapy after surgery [139]. In terms of not only decreased survival, but also the occurrence of metastases, miR-375 has been reported to be a potential prognostic marker of poor outcome and metastasis in HNSCC, and that it may function by suppressing the tumor’s invasive properties [140]. MiRNA can also be used to prognose the risk of recurrence, as high levels of hsa-miR-210 were associated with locoregional disease recurrence and short overall survival [141]. Childs et al. showed that low levels of hsa-miR205 are significantly associated with loco-regional recurrence, independent of the disease severity at diagnosis and treatment. In addition, combined low levels of hsa-miR-205 and hsa-let-7d expression in HNSCC tumors are significantly associated with poor head and neck cancer survival [142]. In terms of the impact on the outcome of chemotherapy, it is worth paying attention to the reports explaining the role that HMGA2 plays in governing genotoxic responses. HMGA2 is associated with enhanced selective chemosensitivity towards the topoisomerase II inhibitor, doxorubicin, in HNSCC [143]. One of the best-studied miRNAs, miR-21, modulates the chemosensitivity of tongue squamous cell carcinoma (TSCC) cells to cisplatin. Since chemoresistance is a huge challenge in tongue cancer management, explaining that miR-21 could modulate the chemosensitivity of cancer cells to cisplatin by targeting PDCD4 presents itself as a promising discovery and potential target for TSCC therapy [144]. A unique group of head and neck cancers consists of HPV-16-mediated cancers, they are more often localized in the oropharynx, and since HPV-infected epithelial cells are more sensitive to chemotherapy this group is characterized by better survival rates [145]. Among that group, the HPV-16-mediated downregulation of Hsa-miR-139-3p may promote oncogenesis in HNC and cervical cancer, and as authors suggest on the basis of this is the viral modulation of host miRNA expression [146]. Based on the miRNA panel, it is also possible to distinguish HPV-positive from HPV-negative HNSCC, and the tests were done in salivary microRNAs. The authors also suggested that the miRNA signature in saliva can even discriminate different stages of HNSCC tumors [147]. The plasma levels of a panel of miRNAs, including miR-142-3p, miR-186-5p, miR-195-5p, miR-374b-5p and miR-574-3p, have been regarded as an HPV-independent prognostic panel for HNSCC patients who were treated with combined radiochemotherapy [148].

### 6.3. Markers of Radiotherapy and Immunotherapy

Radiotherapy (RT) is a significant treatment for patients with head and neck cancer. Despite the advances to improve treatment, many tumors acquire radiation resistance, resulting in poor survival. The differential radiosensitivity has been largely associated with altered cellular DNA damage response mechanisms in HPV-positive HNSCC, and particularly with the signaling and repair of DNA double-strand breaks [149,150]. Since the biological effect of RT differs between patients, there is a strong need for markers that will help to assess the efficacy of therapy, which will allow to classify patients into appropriate groups and assign them personalized treatment, which will significantly increase the chances of higher effectiveness, and perhaps even more importantly allow patients to avoid unnecessary side effects. Unfortunately, while many patients with locally advanced disease are cured with some combination of radiation, and chemotherapy or surgery, others will develop recurrent/metastatic disease and are considered incurable [151]. For such patients, immunotherapy may be an appropriate treatment choice. One of the major advantages of immunotherapy over other forms of systemic cancer therapy is that responses can be quite durable—with clinical benefit sometimes measured in years. Since most patients with metastatic HNSCC do not have a clear tumor-specific target, the discovery of new biomarkers will be essential for improving their outcomes with immunotherapy [152]. The microRNAs suggested as markers in radiotherapy and immunotherapy are presented in Table 2.

### 6.4. Circulating MicroRNAs as a Liquid Biopsy

Liquid biopsy is recently gaining attention for the early diagnosis of cancers, including the HNSCC. It is simple in the procedure and is a relatively quick test examining for circulating tumor cells (CTCs), circulating tumor DNA (ctDNA), circulating miRNAs, and tumor-derived extracellular vesicles (EVs), which are shed from primary tumors and their metastatic sites into the peripheral blood; liquid biopsy seems to be a response to the urgent need for a biomarker that will allow to raise diagnostics to a higher level. Speed, ease of implementation and, above all, non-invasiveness, resulting in a significant reduction in discomfort and risk for the patient, cannot be underestimated in this case [161,162]. However promising this direction may seem, it should be remembered that this approach is innovative and still needs a lot of research, and due to its early stage it tackles the problem of the lack of unified procedures and standardization, already described in earlier chapters [163,164]. However, the potential behind the use of this type of diagnostic and its benefits are pushing research on this topic at a very fast pace, which resulted in the first registration by the US Food and Drug Administration (FDA) of the liquid biopsy test in 2017 [165], which allowed to identify specific changes in single genes only, and soon a modified version based on NGS that can evaluate many different genes at the same time [166]. In regards the use of miRNA as a biomarker in liquid biopsy in HNSCC, the few studies to date seem to be promising. Mazumder et al. showed the potential use of miR-371, miR-150, miR-21 and miR-7d as prognostic markers, and miR-134, miR-146a, miR-338 and miR-371 as metastasis markers in oral cancer [167]. Moreover, the prognostic markers, miR-21 and miR-7d, were also found to be significantly correlated with resistance to chemotherapy. At the same time, the authors point to the still unbeatable difficulties in unravelling the exact regulation of these miRNAs before using them for targeted therapy. In the 2019 meta-analysis, Rapado-González et al. performed a comprehensive synthesis of the possibility of using miRNA in liquid biopsy in the diagnosis of oral squamous cell carcinoma [168]. The author points out that standard biopsy still remains the gold standard, and this situation is mainly due to the lack of validation of miRNA biomarkers and the enormous degree of tumor heterogeneity. Cancer heterogeneity remains one of the greatest problems both in diagnosis and in treatment [169]. Tumor heterogeneity is associated with poor prognosis and outcome, and is one of the leading determinants of therapeutic resistance and treatment failure as well as one of the main reasons for poor overall survival in cancer patients [170]. The very high degree of heterogeneity associated with HNSCC presents an additional challenge in the attempt to use miRNA as a biomarker [171]. On the one hand, an ideal biomarker requires a very high degree of specificity to undoubtedly identify a given type of cancer, but on the other hand, due to the same heterogeneity, a high degree of specificity comes with the risk of not covering all cases [172,173]. Finally worth emphasizing is the use of miRNAs in liquid biopsy not only in peripheral blood, but also in the saliva of patients with HNSCC. In this case, there are also no standards and the research is at a very early stage, but the results seem to be promising and indicate rapid development of this diagnostic branch [174].

## 7. Conclusions and Future Directions

More than a decade of research continues to strengthen the position of miRNAs as a potentially extremely useful biomarker in HNSCC. This is not only in the primary scope of the association with cancer risk modulation, but perhaps even more importantly, in such detailed aspects as predicting the outcomes of chemotherapy, radiotherapy or immunotherapy, and overall survival prognosis. This process is in line with the trend towards the use of miRNAs in medicine, a branch that has already resulted in the implementation of the commercial diagnostic tests, based on microRNA, mentioned earlier. However, as with other neoplastic diseases, and in the use of miRNAs in medical diagnosis in general, the application of HNSCC faces serious problems. The two basic directions in which particular emphasis should be placed include the unquestionable establishment of connections between the aspect under study and a particular miRNA, and the standardization of the diagnostic procedures. In the first aspect, reports on the non-specificity of miRNA as a biomarker return inconclusive results of correlation with clinical events, and sometimes conflicting research results indicate the need for a final determination of the role of miRNA in the pathogenesis, development and response to cancer treatment. The problem of tumor heterogeneity is also important here, which requires a very delicate approach and finding the perfect compromise between the sensitivity and specificity of the biomarker, and the risk of obtaining false negative results. In the second aspect, which also has an undeniable impact on the problems described in the first point, methodological problems and the lack of unambiguous standardization criteria make it impossible to validate miRNA as a biomarker in HNSCC and implement it for use in clinical practice. The creation of standardized guidelines and protocols is a solution leading to both obtaining reliable research results and a contribution to clinical implementation. The creation of a database compiling the existing knowledge on the use of miRNAs in diagnostics, treatment and prognosis would be extremely helpful in such standardization. Currently, there are several projects grouping the existing data, but none are at the stage of clinical implementation. It seems that we are at the stage of emerging a leader in this field, who will play such a role in the future. Currently, one can use databases such as miR2Disease (manually curated database for microRNA deregulation in human disease) [175], SomamiR 2.0 (a database of cancer somatic mutations altering microRNA–ceRNA interactions) [176], dbDEMC (a database of differentially expressed miRNAs in human cancers) [177], miRmine (a database of human miRNA expression profiles) [178] or TANRIC (an interactive open platform to explore the function of lncRNAs in cancer) [179]. Over time, some of these databases have become obsolete, e.g., miR2Disease offers only about 30 entries about all types of HNSCC, all from before 2010, while others are dynamically developing—SomamiR 2.0 accumulates almost 300 entries only for the lower third part of esophagus squamous cell carcinoma. Moreover, although there is no database dedicated exclusively to HNSCC, with the data offered in existing sources for all types of cancer, you have access to almost all miRNA alterations. However, it should be emphasized once again that the issue of standardization of these data remains unresolved, and the fact that at the present stage of their development, the data contained therein can be used primarily for scientific research and not for clinical implementation. However, both of the abovementioned main aspects (lack of standardization and no compelling data), while currently problematic, are not unsolvable and, although they require a lot of work, they cannot rule out the potential benefits of implementing miRNA as a biomarker in HNSCC. Obtaining a fast, reliable, standardized and non-invasive diagnostic path for HNSCC patients will certainly bring great benefits and is worth further work.

## Figures and Tables

**Table 1:** Selected genes and their action mechanism in HNSCC.

Oncogenes MiRNAs	Affected Gene	Molecular Mechanism	Action Mode	Ref
miR-125a	p53	miR-125a enhances cell proliferation, migration, invasion	Gene expression	[117]
miR-134	PDCD7	miR-134 reduces E-cadherin expression by suppressing PDCD7	Gene expression	[118]
miR-134	WWOX	miR-134 suppresses WWOX	Suppressor inhibition	[121]
miR-196b	PCDH-17	miR-196b promotes cell proliferation, migration, and invasion abilities by inhibiting PCDH-17	Suppressor inhibition	[127]
miR-106A-5p	BTG3	miR-106A-5p inhibits autophagy and activates MAPK signaling by targeting BTG3	Signal transduction	[128]
**Suppressors MiRNAs**	**Affected Gene**	**Molecular Mechanism**	**Action Mode**	**Ref**
let-7i	ARID3B	let-7i inhibition enhances ARID3B expression and activates the expression of POU5F1, NANOG, and SOX2	Gene expression	[129]
let-7c	CXCL8	let-7c inhibition enhances stemness and radio-/chemoresistance by suppressing CXCL8	Signal transduction	[130]
miR-101	EZH2	miR-101 inhibits EZH2 and suppresses metastasis and EMT	Signal transduction	[131]
miR-101	CDK8	miR-101 inhibits CDK8 expression and subsequently suppresses Wnt/β-catenin signaling and tumorigenesis	Signal transduction	[132]
miR-124	STAT3	miR-124 inhibits tumor growth and metastasis by suppressing STAT3	Signal transduction	[133]

**Table 2:** MicroRNA as radiotherapy and immunotherapy markers.

Radiotherapy			
MicroRNA	Regulation	Potential Use	Ref
miR-186-5p, miR-374b-5p, and miR-574-3p	None	Shorter progression-free or overall survival rate in RT patients	[148]
miR-296-5p	Upregulated	Resistance to radiotherapy marker	[153]
miR-93, miR-200a	Upregulated	Treatment monitoring post-radiation	[154]
miR-324-3p, miR-93-3p, miR-4501	Downregulated	Resistance to radiotherapy marker	[155]
miR-371a-5p, miR-34c-5p, miR-1323	Upregulated	Resistance to radiotherapy marker	[155]
miR-150, miR-1254, miR-16, miR-29b	Upregulated	Resistance to radiotherapy marker	[156]
miR-141, miR-18b, miR-301a	Downregulated	Resistance to radiotherapy marker	[157]
**Immunotherapy**			
**MicroRNA**	**Regulation**	**Potential Use**	**Ref**
miR-199a-3p, miR-21-5p, miR-28-5p	Downregulated	Immunotherapy predicting marker	[158]
miR-200c-3p, miR-21-5p, miR-28-5p	Downregulated	Anti PD-1/PD-L1 treatment response marker	[159]
let-7 family	Downregulated	Immunotherapy predicting marker	[160]

## Data Availability

Not applicable.
